# Circular RNA circVAMP3 promotes aerobic glycolysis and proliferation by regulating LDHA in renal cell carcinoma

**DOI:** 10.1038/s41419-022-04863-0

**Published:** 2022-05-07

**Authors:** Jun Li, Qian Zhang, Yupeng Guan, Dingzhun Liao, Donggen Jiang, Haiyun Xiong, Hengji Zhan, Jun Pang

**Affiliations:** 1 Department of Urology, Kidney and Urology Center, The Seventh Affiliated Hospital, Sun Yat-sen University, Shenzhen, 518107 China; 2 Department of Rehabilitation Medicine, The Seventh Affiliated Hospital, Sun Yat-sen University, Shenzhen, 518107 China; 3 Department of Pathology, The Seventh Affiliated Hospital, Sun Yat-sen University, Shenzhen, 518107 China; 4 Department of Urology, Sun Yat-sen Memorial Hospital, Sun Yat-sen University, Guangzhou, 510220 China

**Keywords:** Cancer metabolism, Cell growth

## Abstract

Metabolic dysfunction is seen in cancer cells where increased glycolysis provides energy for growth. Circular RNAs (circRNAs) are thought to assist in glucose metabolism and the switch to glycolysis. Through screening, we found that circVAMP3 was necessary for both glycolytic and proliferative activities in renal cell carcinoma (RCC). Furthermore, circVAMP3 expression was elevated in RCC patients in correspondence with TNM stage. Mechanistically, circVAMP3 was observed to interact directly with lactate dehydrogenase A (LDHA) and modulate its activity. The circVAMP3–LDHA interaction facilitated LDHA phosphorylation at tyrosine 10 (Y10) catalyzed by the upstream kinase fibroblast growth factor receptor type 1 (FGFR1). Therefore, this study reveals a novel molecular mechanism by which circVAMP3 promotes glycolysis and proliferation through regulating the enzymatic activity of glycolytic enzyme, suggesting that circVAMP3 may represent an RCC biomarker and treatment target.

## Introduction

Renal cell carcinoma (RCC) is an aggressive neoplasm of the genitourinary system, and its morbidity and mortality have been increasing over the past decade [1]. The clear-cell RCC subtype (ccRCC) makes up nearly 70–75% of all RCC cases [2]. Among the current methods for RCC treatment, radical/partial nephrectomy remains the most effective therapy for early-stage cases. Early RCC is hard to detect and patients with locally advanced or metastatic disease exhibit a very poor prognosis of a less than 12% five-year survival rate [3], despite significant advances in treating RCC using targeted therapies and immunotherapy. However, such therapeutic effects are still limited [4]. This scenario urges us to further investigating the key mechanisms underlying RCC pathogenesis and identify new diagnostic and therapeutic targets.

Metabolic alterations are commonly observed in the development of human cancers, including RCC [5]. In particular, cancer cells tend to have elevated levels of glycolysis, reducing glucose to lactate. Glycolysis may be anaerobic or aerobic, and the latter, as aerobic glycolysis or the Warburg effect, commonly occurs in cancer cells. This allows the production of increased energy and materials for the increased biosynthetic levels. Abnormal expression or acquired genetic mutations of many metabolic enzymes can result in metabolic disorders and tumorigenesis [6]. As one of key metabolic enzymes in the glycolytic pathway, lactate dehydrogenase A (LDHA) catalyzes reduction of pyruvate to lactate and is essential for the reduction of pyruvate to lactate and might be linked to tumorigenesis and malignant progression by driving the Warburg effect [7]. It has been found that LDHA is strongly expressed in many human cancers and has been linked to poor patient outcomes, as seen in pancreatic [8], colorectal [9], and breast cancer [10], as well as RCC [11]. Moreover, genetic silencing of LDHA suppresses tumor growth and induces apoptosis of tumor cells [12, 13]. All these findings suggest that LDHA may be a possible target molecule for cancer treatment.

Recently, many circular RNAs (circRNAs) have been shown to modulate cancer cell metabolism. For example, circMAT2B promoted tumor progression and PKM2-mediated aerobic glycolysis in hepatocellular carcinoma by sponging miR-338-3p [14]. Furthermore, circCUX1 was reported to bind to EWSR1, transactivating MAZ, promoting the Warburg effect, and eventually contributing to neuroblastoma progression [15]. However, the relationship between circRNAs and glycolysis in RCC is not known.

Here, we discovered a circRNA derived from the VAMP3 gene, circVAMP3, which plays a critical role both in glucose metabolism and cell viability in RCC cells. Furthermore, circVAMP3 was found to be strongly expressed in RCC tumors, with the elevated levels correlated with advanced TNM stage. Importantly, this is the first circRNA that has been found to interact directly with LDHA and thus modulate its activity. The circVAMP3–LDHA binding facilitated LDHA phosphorylation at tyrosine 10 (Y10) catalyzed by the upstream kinase FGFR1, thus improving LDHA activity and promoting glycolysis to drive the progression of RCC. Therefore, circVMAP3 may represent a useful biomarker and target for treating RCC.

## Results

### Identification of circVAMP3 as a metabolism-related circRNA

**Fig. 1:**
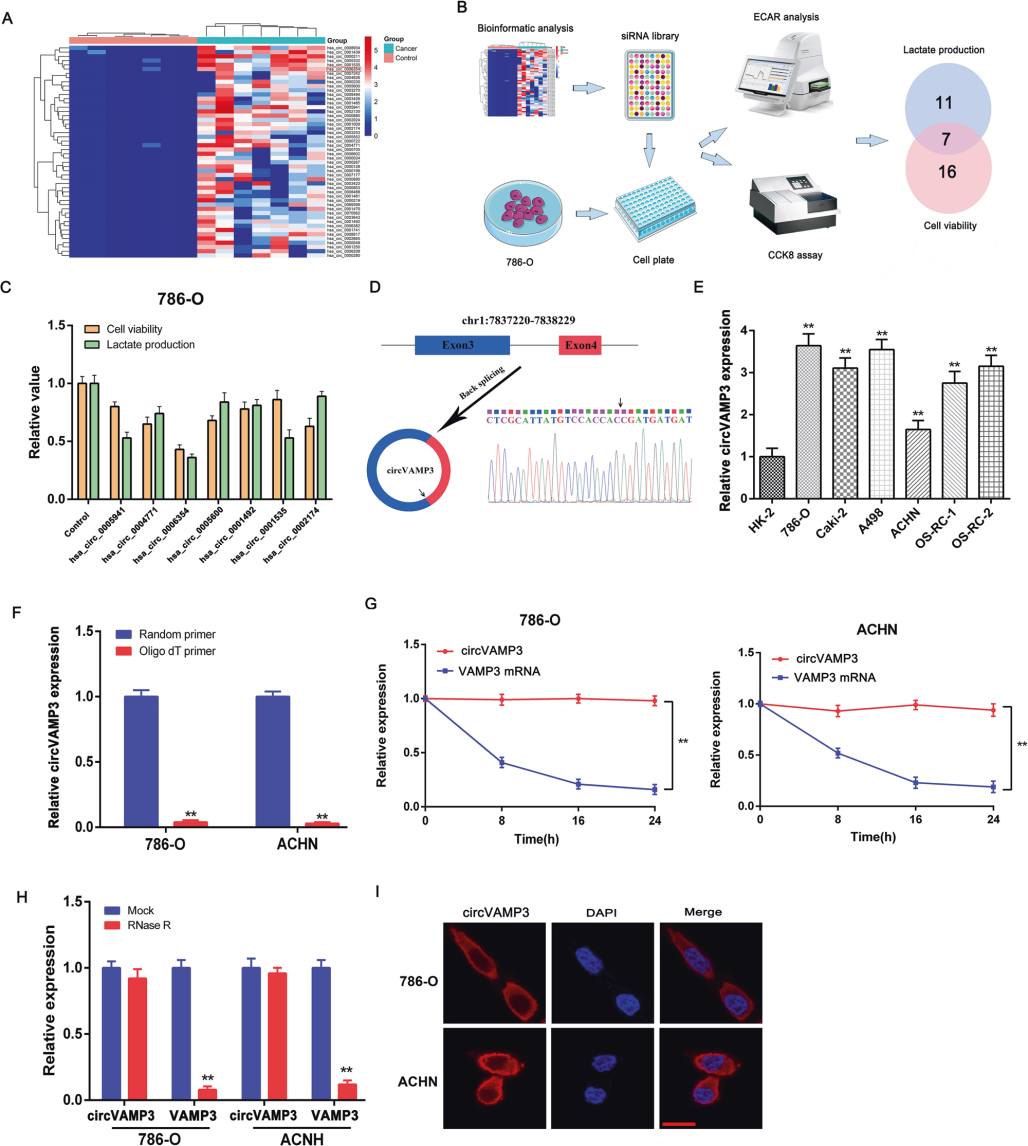
Identification of circVAMP3 as a metabolism-related circRNA. **A** Top-50 circRNAs that are highly expressed in RCC according to the GEO dataset (GSE108735). **B** Flowchart for identification of circRNAs potentially involved in both glucose metabolism and cellular viability. **C** Seven circRNAs regulated both lactate production and cell viability in 786-O cells. **D** Scheme illustrating the production of circVAMP3 and sequencing analysis of back-splicing junction in circVAMP3. **E** Expression of circVAMP3 determined by qRT-PCR in RCC cell lines (786-O, Caki-2, A498, ACHN, OS-RC-1, and OS-RC-2) and in the normal human proximal tubule epithelial cell line (HK-2). **F** qRT-PCR of circVAMP3 in the reverse-transcribed products using random primers or oligo-dT primers. **G** Relative RNA levels assessed by qRT-PCR after actinomycin-D application. **H** RNA levels in cells with and without RNase R treatment. **I** FISH localization of circVAMP3. circVAMP3 was labeled with Cy3. Scale bar, 20 μm. The results are presented as the mean ± SD. *P < 0.05; **P < 0.01.

We used microarray data from the GEO dataset GSE108735 to identify candidate circRNAs relevant for RCC, and further normalized and analyzed using GEO2R after applying log2 transformation. The 50 top-ranking circRNAs were used to build an siRNA library (Fig. 1A). To identify circRNAs potentially involved in glucose metabolism, the library was transfected into the 786-O RCC cell line, and viability and lactate production were measured. This identified 16 candidate circRNAs for proliferation, 11 for lactate production, and 7 that might promote cell viability and glucose metabolism (Fig. 1B). Of these seven circRNAs, knockdown of hsa_circ_0006354 was found to significantly decrease both viability and lactate production (Fig. 1C). According to the circBase dataset, hsa_circ_0006354 is derived from the VAMP3 gene on chromosome 1, the result of backsplicing of exons 3 and 4. For simplicity, we refer to this circRNA as circVAMP3. Sanger sequencing was used to confirm the back-splicing junctions of circVMAP3 (Fig. 1D). The six RCC cell lines (786-O, Caki-2, A498, ACHN, OS-RC-1, and OS-RC-2) showed elevated circVAMP3 expression compared with the normal HK-2 cells (Fig. 1E). As circRNAs lack 3′ polyadenylated tails, we investigated the presence of circVAMP3 with random or oligo-dT primers, observing that almost no circVAMP3 was detectable when oligo-dT primers were applied (Fig. 1F). The Actinomycin-D assay confirmed that circVAMP3 had a longer half-life than VAMP3 mRNA (Fig. 1G). It was also found to be more resistant to RNase R treatment (Fig. 1H). FISH investigation of the subcellular location of circVAMP3 in 786-O and ACHN cells showed that it was located in the cytoplasm (Fig. 1I).

### CircVAMP3 is upregulated in human RCC tissues and is positively associated with TNM stage

**Fig. 2:**
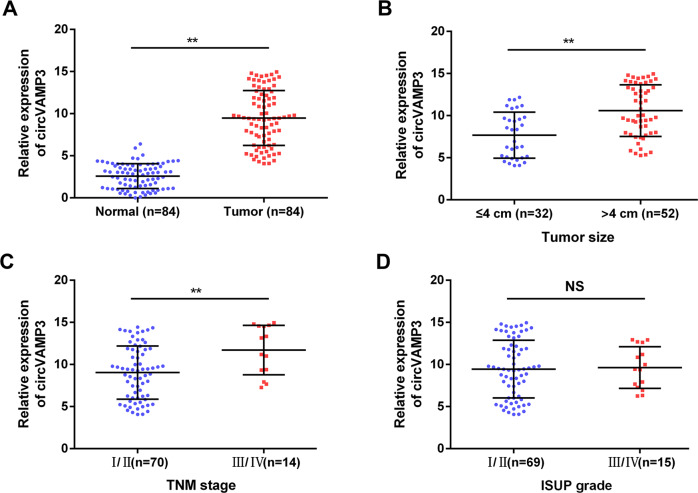
CircVAMP3 is upregulated in human RCC tissue and is positively correlated with TNM stage. **A** Relative expression of circVAMP3 in 84 paired RCC and normal tissues measured by qRT-PCR. **B** CircVAMP3 levels detected by qRT-PCR in the tumors ≤4 cm (*n* = 32) and å 4 cm (*n* = 52). **C** CircVAMP3 levels determined by qRT-PCR in TNM I/II stage (*n* = 70) versus TNM III/IV stage (*n* = 14). **D** CircVAMP3 expression levels were examined by qRT-PCR in the Fuhrman I/II-grade group (*n* = 69) versus in the Fuhrman III/IV-grade group (*n* = 15). The results are presented as the mean ± SD. *P < 0.05; **P < 0.01; NS nonsignificant.

Using qRT-PCR, we investigated the expression of circVAMP3 in 84 pairs of RCC and normal specimens. The results showed that circVAMP3 was significantly upregulated in RCC tissue (Fig. 2A). Furthermore, we analyzed whether circVAMP3 levels in RCC were related to clinical and pathological traits. The circVAMP3 levels were found to be elevated in tumors å 4 cm (*n* = 32) than in tumors ≤4 cm (*n* = 52), as well as higher in the TNM III/IV stage (*n* = 14) than in TNM I/II-stage specimens (*n* = 70) (Fig. 2B, C). However, the circVAMP3 expression levels had no significant difference among different ISUP grades (Fig. 2D).

### CircVAMP3 promotes glycolysis in RCC cells

**Fig. 3:**
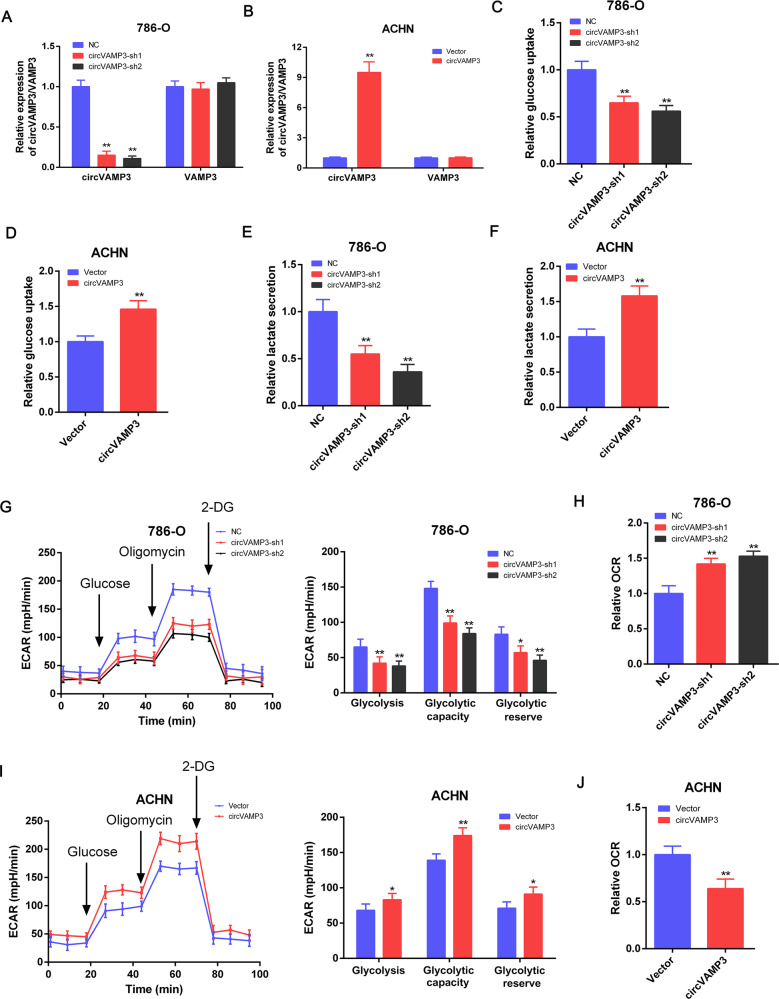
CircVAMP3 promotes glycolysis in RCC cells. **A** qRT-PCR measurement of circVAMP3 and VAMP3 mRNA in 786-O cells after stable transfection of circVAMP-sh1/2 or NC. **B** qRT-PCR analysis of circVAMP3 and VAMP3 mRNA in ACHN cells after stable transfection of circVAMP3 or vector. **C** Glucose-uptake levels after circVAMP3 knockdown. **D** Glucose-uptake levels after circVAMP3 overexpression. **E** Lactate levels after circVAMP3 knockdown. **F** Lactate levels after circVAMP3 overexpression. **G** The extracellular acidification rate (ECAR) was analyzed using the “Seahorse analyzer” after circVAMP3 knockdown. The values of glycolysis, glycolysis capacity, and glycolytic reserve were calculated. **H** The “relative oxygen consumption rate (OCR)” was measured using the “Seahorse analyzer” in 786-O cells with circVAMP3 knockdown. **I** ECAR after circVAMP3 overexpression. The values of glycolysis, glycolysis capacity, and glycolytic reserve were calculated. **J** OCR after circVAMP3 overexpression. The results are presented as the mean ± SD. *P < 0.05; **P < 0.01.

To examine the actions of circVAMP3, knockdown and overexpression experiments were used. We were able to stably knock down circVAMP3 in 786-O cells and overexpress it in ACHN cells by lentivirus transfection. The efficiencies of the knockdowns and overexpression were assessed by qRT-PCR (Fig. 3A, B). The levels of VAMP3 mRNA were not affected by circVAMP3 changes (Fig. 3A, B). Among all kinds of renal cancer cell lines (786-O, Caki-2, A498, ACHN, OS-RC-1, and OS-RC-2) in our study, the qRT-PCR results indicated that the relative expression of circVAMP3 was the highest in 786-O cells and the lowest in ACHN cells (Fig. 1E). Therefore, we selected 786-O cells to perform loss-of-function experiments, and ACHN cells to carry out gain-of-function experiments, which may better perform the biological functions of circVAMP3.

To further verify the effect of circVAMP3 on glycolysis, we measured glucose and lactate levels, as well as glycolysis rate, in knockdown and overexpressing RCC cells. Both glucose and lactate levels were lower in the knockdown 786-O cells, while overexpression resulted in significant increases in both in ACHN cells (Fig. 3C–F). Furthermore, both glycolysis and ECAR were assessed by the Seahorse Analyzer. This process is shown in Fig. 3G. The glycolytic reserve was estimated using 2-deoxy-D-glucose (2-DG) to block glycolysis. The results confirmed that circVAMP3 knockdown reduced the ECAR in 786-O cells, while upregulation of circVAMP3 increased ECAR in ACHN cells (Fig. 3G, I). Glycolysis, the glycolysis capacity, and glycolytic reserve were markedly lowered by circVAMP3 knockdown, but were increased by circVAMP3 overexpression (Fig. 3G, I). Measurement of the OCR, a measure of oxidative phosphorylation, showed that it was raised in the knockdown cells, while overexpression of circVAMP3 had the opposite effect (Fig. 3H, J). These findings indicate that circVAMP3 promotes glycolysis and reduces oxidative phosphorylation.

### CircVAMP3 promotes proliferation of RCC cells

**Fig. 4:**
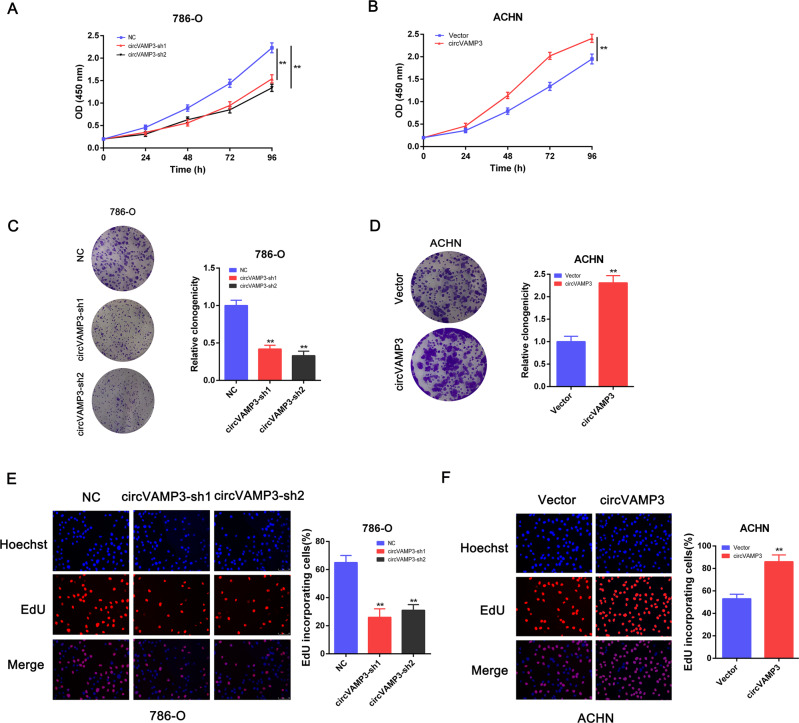
CircVAMP3 promotes proliferation in RCC cells. **A** CCK-8 assay was performed to analyze cell viability in 786-O cells with circVAMP3 knockdown. **B** CCK-8 assay was performed to analyze cellular viability after circVAMP3 overexpression. **C** Micrographs (left panel) and relative quantification (right panel) of colony-formation assay after circVAMP3 knockdown. **D** Micrographs (left panel) and relative quantification (right panel) of colony-formation assay after circVAMP3 overexpression. **E** Micrographs (left panel) and quantification (right panel) of EdU-incorporated cells in 786-O cells with circVAMP3 knockdown. **F** Micrographs (left panel) and quantification (right panel) of EdU-incorporated cells after circVAMP3 overexpression. The results are presented as the mean ± SD. *P < 0.05; **P < 0.01.

To further investigate the role of circVAMP3 in RCC, CCK-8, EdU, and colony-formation assays were conducted. It was apparent that reducing circVAMP3 suppressed proliferation in RCC cells (Fig. 4A–F), while overexpression had the opposite effect (Fig. 4A–F). These findings suggest that circVAMP3 functions as an oncogene in RCC cells.

### CircVAMP3 interacts directly with LDHA and promotes LDHA activity in RCC cells

When miRNA forms RISC (RNA-induced silencing complex), it is wrapped by AGO protein, mainly AGO2. In other words, by pulling down AGO2 protein, the miRNA bound to it can be pulled down, so the RISC-bound circRNA can also be pulled down. To evaluate whether cytoplasmic circVAMP3 acts as a miRNA sponge, we performed an AGO2 RIP assay, observing that circVAMP3 was not able to bind to AGO2 (Supplementary Fig. S1). We thus discarded the hypothesis that circVAMP3 acts as a miRNA sponge.

**Fig. 5:**
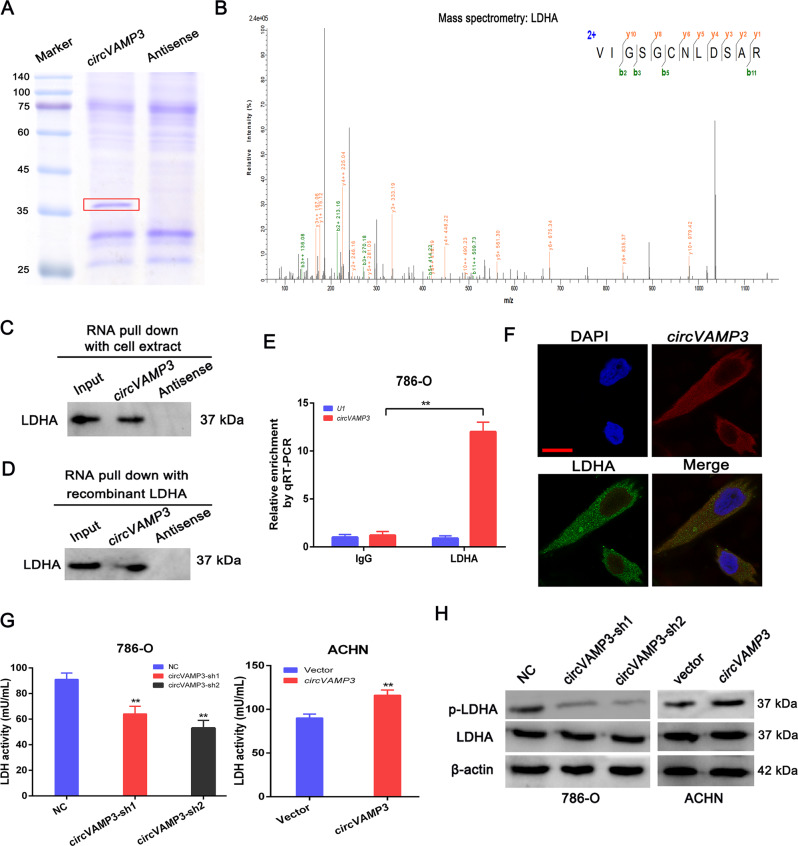
CircVAMP3 directly binds to glycolytic enzyme LDHA and elevates its activity. **A** RNA pull-down assay using specific biotin-labeled circVAMP3 (sense) and control (antisense) probes in 786-O cells, followed by coomassie brilliant blue staining. The red box indicates an obvious 35~45-KDa band. **B** MS identification of the circVAMP3-interacting protein as LDHA. **C**, **D** RNA pulldown and western blotting with 786-O cell extract (**C**) or purified recombinant LDHA (**D**) evaluated the direct interaction between circVAMP3 and LDHA. **E** qRT-PCR analysis of RNA enrichment in the RIP assay using the anti-LDHA antibody in 786-O cells. lgG: nonspecific control antibody; U1: negative control. **F** The cellular localizations of circVAMP3 and LDHA were assessed by FISH and immunofluorescence in 786-O cells. Scale bar: 20 μm. **G** LDH activities after circVAMP3 knockdown or overexpression. **H** Western blotting of p-LDHA (Y10) and LDHA expression in RCC cells with circVAMP3 knockdown or overexpression. The results are presented as the mean ± SD. *P < 0.05; **P < 0.01.

Recent evidence suggests that circRNAs act as modulators through protein interaction [16]. We used RNA pulldown to investigate this hypothesis, using biotinylated probes targeting the circVAMP3 back-spliced sequence. These pulldowns with biotinylated circVAMP3 and antisense controls produced a clear band between 35 and 45 kDa (Fig. 5A), which was identified by MS as LDHA (Fig. 5B). LDHA is an important modulator of glycolysis, where it is responsible for reversibly converting pyruvate to lactate during the final glycolytic step. Furthermore, western blotting of the pulled-down circVAMP3-enriched proteins indicated that circVAMP3 could interact with endogenous LDHA in RCC cells (Fig. 5C) as well as with the recombinant LDHA protein (Fig. 5D), indicating direct binding between circVAMP3 and LDHA. RIP also showed that circVAMP3 was enriched by LDHA protein, further verifying binding between circVAMP3 and LDHA (Fig. 5E). In addition, confocal microscopy of circVAMP3 FISH and LDHA immunostaining showed cytoplasmic colocalization of circVAMP3 and LDHA in RCC cells (Fig. 5F).

Next, we studied the consequences of circVAMP3/LDHA binding. The knockdown of circVAMP3 reduced LDH activity in 786-O cells, and overexpression of circVAMP3 enhanced LDH activity in ACHN cells (Fig. 5G). LDHA activity is known to be influenced by phosphorylation [17], therefore, we hypothesized that circVAMP3 might modify phosphorylation of LDHA. As shown in Fig. 5h, silencing circVAMP3 significantly reduced LDHA phosphorylation at tyrosine 10 (Y10), and overexpression of circVAMP3 enhanced the phosphorylation level. These results indicated that circVAMP3 modulates LDHA enzymatic activity potentially through modifying phosphorylation at Y10.

### CircVAMP3 modulates LDHA through FGFR1

**Fig. 6:**
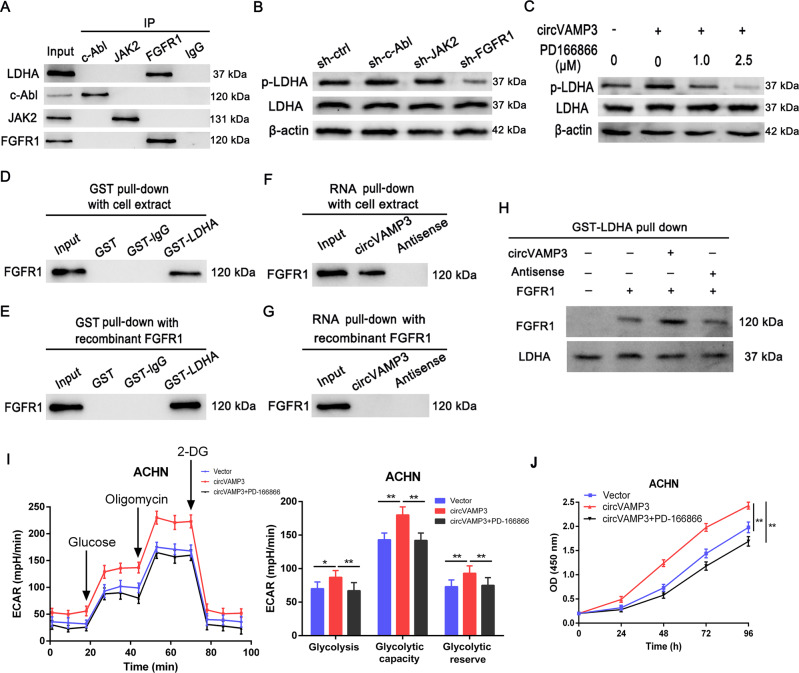
CircVAMP3 regulates LDHA phosphorylation by FGFR1. **A** Co-IP analysis of the interaction between endogenous LDHA with c-Abl, JAK2, or FGFR1 in 786-O cells. **B** The levels of p-LDHA (Y10) and LDHA were examined by western blotting in 786-O cells after silencing the expression of c-Abl, JAK2, or FGFR1. **C** Western blotting of p-LDHA (Y10) and LDHA expression in the cell lysates. ACHN cells overexpressing circVAMP3 were treated with different concentrations of FGFR1 inhibitor PD-166866 for 24 h. **D**, **E** GST pulldown and western blotting with 786-O cell extract (**D**) or purified recombinant FGFR1 (**E**) assessed the direct interaction between GST-LDHA and FGFR1. lgG: nonspecific control antibody. **F**, **G** RNA pulldown and western blotting with 786-O cell extract (**F**) or purified recombinant FGFR1 (**G**) evaluated the indirect interaction between circVAMP3 and FGFR1. **H** GST-tagged recombinant LDHA pulldown with recombinant FGFR1, followed by the addition of circVAMP3 or antisense circVAMP3. **I** ECAR of circVAMP3-overexpressing cells incubated with the FGFR1 inhibitor PD-166866 (2.5 μM). The values of glycolysis, glycolysis capacity, and glycolytic reserve were calculated. **J** Cell proliferation was analyzed by CCK-8 assay. For rescue experiments, circVAMP3-overexpressing cells were treated with the FGFR1 inhibitor PD-166866 (2.5 μM). The results are presented as the mean ± SD. *P < 0.05; **P < 0.01.

It was reported that several oncogenic tyrosine kinases phosphorylated LDHA at Y10 in various cancers, such as FGFR1 in lung cancer, BCR-Abl in chronic myelogenous leukemia, and JAK2 in human erythroleukemia [18]. Thus, we suspected whether these three tyrosine kinases were responsible for LDHA Y10 phosphorylated in RCC. We first performed Co-IP to analyze the interaction of LDHA with these three tyrosine kinases. The results indicated that LDHA only interacted with FGFR1 in RCC cells (Fig. 6A). Furthermore, when we silenced the expression of these three genes, only FGFR1 knockdown reduced the LDHA Y10 phosphorylation level, while the other two tyrosine kinases had no effect on p-LDHA (Fig. 6B). Therefore, FGFR1 was confirmed to be responsible for LDHA Y10 phosphorylation in RCC cells. In addition, circVAMP3 overexpression enhanced the Y10 phosphorylation of LDHA, which was blocked in a dose-dependent manner by the FGFR1 inhibitor PD-166866, suggesting that circVAMP3 regulates Y10 phosphorylation of LDHA through FGFR1 (Fig. 6C).

To identify whether FGFR1, LDHA, and circVAMP3 form a protein–RNA complex, we used GST pulldown, observing that FGFR1 interacts directly with LDHA (Fig. 6D, E). Moreover, this was supported by the RNA pulldown showing that circVAMP3 was able to interact with endogenous FGFR1 (Fig. 6F) but not with recombinant FGFR1 protein (Fig. 6G), indicating that circVAMP3 could interact with FGFR1 but not directly. In addition, we examined whether the binding of circVAMP3 could affect the interactions between LDHA and FGFR1. The GST-LDHA pull-down assay indicated that the addition of circVAMP3 instead of the antisense circVAMP3 increased the direct binding between FGFR1 and LDHA (Fig. 6H). These results suggested that these three molecules form a protein–RNA complex, the interaction between circVAMP3 and FGFR1 was mediated by LDHA.

Next, we investigated the impact of FGFR1/circVAMP3/LDHA axis on the glycolytic and proliferative ability of RCC cells. As shown in Figs. 6I, J, circVAMP3 overexpression increased both glycolysis and proliferation in ACHN cells, and treatment with the FGRF1 inhibitor PD-166866 abolished these effects. These findings indicate that circVAMP3 promotes glycolysis and proliferation in RCC cells by modulating LDHA through FGFR1.

### CircVAMP3 promotes RCC cell growth in vivo

**Fig. 7:**
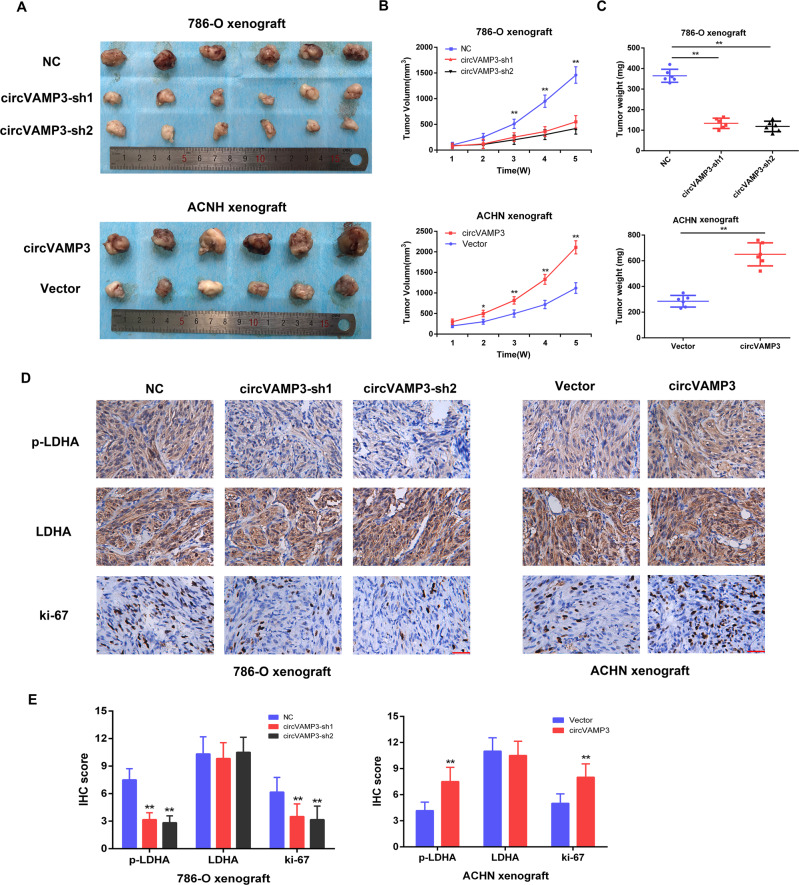
CircVAMP3 promotes tumor growth of RCC cells in vivo by phosphorylation of LDHA. **A** Images of dissected xenograft tumors, showing circVAMP3-overexpressing, silenced, and control cells (*n* = 6 per group). **B** The tumor volume of xenografts in nude mice was calculated weekly for five weeks. **C** The final tumor weight was recorded after dissecting the tumors. **D** IHC staining of p-LDHA (Y10), LDHA, and ki-67 in tumor tissues from the different groups. Scare bar: 50 μm. **E** Summary and statistical analysis of p-LDHA (Y10), LDHA, and ki-67 IHC score in the indicated groups. The results are presented as the mean ± SD. *P < 0.05; **P < 0.01.

To examine the oncogenic action of circVAMP3 in vivo, we injected nude mice with circVAMP3-knockdown 786-O cells and circVAMP3-overexpressing ACHN cells (6 animals per group). After five weeks, it was apparent that tumor growth was slower and tumor weights were lower with the knockdown cells, whereas circVAMP3 overexpression had the opposite effects (Fig. 7A–C). Furthermore, IHC showed decreased levels of p-LDHA and ki-67 in the circVAMP3-knockdown group (Fig. 7D), with significant IHC scores for both (Fig. 7E), whereas circVAMP3 overexpression increased p-LDHA and ki-67 expression levels (Fig. 7D, E). Meanwhile, the expression level of LDHA protein was almost unchanged whether the expression of circVAMP3 was downregulated or upregulated (Fig. 7D, E). These findings indicate that circVAMP3 promotes tumor growth through enhancing LDHA phosphorylation in vivo.

## Discussion

Metabolic changes, especially activation of glycolysis, are typical of neoplastic cells. Raised levels of glycolysis, seen by elevated consumption of glucose and production of lactate, are seen in many tumor cell types, and provide both energy and materials to support tumor growth. High rates of glycolysis are related to poor outcomes in RCC patients, and have been associated with tumor aggression, including proliferation and metastasis [19]. Recent studies have implicated circRNAs in glycolytic regulation, but most of these circRNAs appear to act by regulating gene expression [20]. Here, we showed that circVAMP3 promotes proliferation and glycolysis by influencing LDHA activity without affecting its gene expression. Interestingly, this is the first circRNA found to regulate and interact directly with LDHA, which enriches our knowledge of the modulatory actions of circRNA molecules, especially in cellular metabolism.

The roles of circRNAs in modulating tumor development and progression are poorly understood. The most widely reported function of circRNA is that of sponging miRNAs to regulate target gene expression, resulting in an effect of promoting or inhibiting cancer [21]. However, recent reports have suggested that circRNA may also act through other means, including directly binding to protein [22] and translating peptides [23]. Here, we ruled out the role of circVAMP3 as a miR sponge, observing that circVAMP3 functions by direct protein interaction with LDHA in RCC cells. However, the potential ability of circVMAP3 to translate peptides still requires further exploration.

LDHA is responsible for catalyzing the production of lactate from pyruvate and its activity is necessary to glycolytic regulation. Differences in posttranslational modifications have been suggested to regulate the enzymatic activity of LDHA in human cancers [24]. These studies indicate that LDHA activity is mainly modulated by phosphorylation at tyrosine 10 (Y10) [17, 25], which supports the current findings. Here, we observed increased Y10 phosphorylation in circVAMP3-overexpressing RCC cells, which was accompanied by enhanced LDHA activity. In addition, acetylation of lysine 5 on LDHA has been reported to reduce its activity in pancreatic cancer [26]. Investigation of the potential crosstalk between these posttranslational modifications and their effects on LDHA enzyme function, especially in relation to cancer, would be informative.

It has been reported that several oncogenic tyrosine kinases phosphorylated LDHA at Y10 in various cancers, such as FGFR1 in lung cancer, BCR-Abl in chronic myelogenous leukemia, and JAK2 in human erythroleukemia [17]. In this report, we confirmed that the effect of upstream kinases in facilitating LDHA Y10 phosphorylation is specific to FGFR1, indicating that the tyrosine kinase responsible for LDHA Y10 phosphorylation is cancer cell-type specific.

There are some limitations in this study. In terms of clinical specimens, we only determined the link between circVAMP3 levels and patient clinical parameters, and failed to identify the predictive value of circVAMP3 for patient clinical prognosis, which was mainly due to the lack of extended follow-up data. In addition, the glucose metabolism levels were not detected in our animal models, which needs more efforts to overcome the difficulties.

**Fig. 8:**
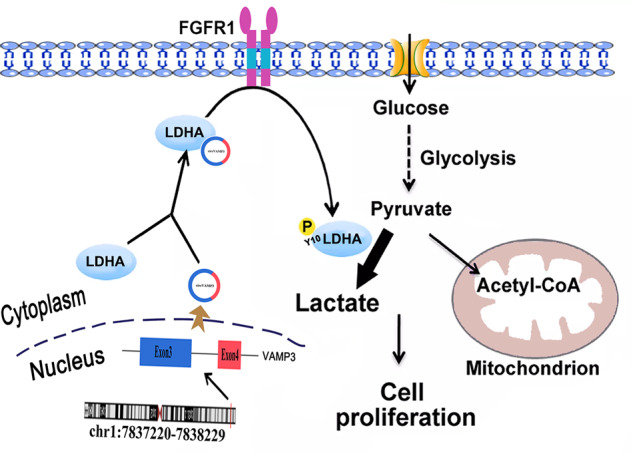
Graphical abstract of circVAMP3-mediated oncogenic mechanism in RCC progression. The newly identified circVAMP3 directly binds to LDHA, elevates its Y10 phosphorylation through the upstream kinase FGFR1, and then enhances the enzymatic activity of LDHA, contributing to elevated glycolytic levels and promoting proliferation in RCC cells.

In summary, we identified oncogenic circVMAP3 and explored its role in regulating glycolytic and proliferative functions in RCC cells. The findings suggest that circVAMP3 is strongly expressed in RCC and correlates with the TNM stage. Mechanistically, circVAMP3 directly binds to LDHA, elevates its Y10 phosphorylation through the upstream kinase FGFR1, and then enhances the enzymatic activity of LDHA, contributing to elevated glycolytic levels and promoting proliferation in RCC cells, as shown in Fig. 8. This study may provide strategies targeting circRNAs and cancer metabolism for cancer treatment.

## Materials and methods

### Cells

A human renal proximal tubular epithelial cell line (HK-2) and the human RCC cell lines (786-O, Caki-2, A498, ACHN, OS-RC-1, and OS-RC-2) were acquired from the ATCC. In RPMI 1640 milieu (Gibco, China) supplemented with1% streptomycin/penicillin and 10% FBS, the RCC lines were grown. HK-2 cells were cultivated in DMEM (Gibco, China) by employing the same FBS and antibiotic concentrations at 37 °C with 5% CO_2_.

### ccRCC patients and samples

The patients included 84 patients with ccRCC, who had undergone surgical resection without radiotherapy or chemotherapy between 2018 and 2021, at the Seventh Affiliated Hospital of Sun Yat-sen University (Shenzhen, China). Tumor samples as well as adjoining control tissue samples were collected and frozen before extracting total RNA with a “nucleic acid isolation kit” (ThermoFisher, USA). The tumor staging relied on the 8th TNM system from American Joint Committee on Cancer (AJCC). The tumor pathological grading system was determined in accordance with the 2012 International Society of Urological Pathology Consensus Conference (ISUP Grade) [27]. The contributors presented the letter of satisfaction and the research was confirmed through the Medical Ethics Committee of the Seventh Affiliated Hospital of Sun Yat-sen University.

### RNA/gDNA extraction, RNase R treatment, cDNA synthesis, and qRT-PCR

The extraction of total RNA was obtained from cells by TRIzol (Invitrogen, USA), and genomic DNA (gDNA) was extracted employing the “MiniBEST Universial Genomic DNA Extraction Kit” (Takara, Japan) following supplied protocols. In the case of RNase R processing, the same amount of total RNA (2 μg) was treated at 37 °C for 30 min with/without 3 U/μg RNase R (Epicenter Technologies, USA) and the purification of product RNAs was done using an “RNeasy MinElute Cleanup Kit” (Qiagen, Germany). cDNA was acquired implementing a “PrimeScript RT Kit” (Takara, Japan) with random or oligo (dT) primers. Real-time polymerase chain reaction (qRT-PCR) was executed on an Applied Biosystems StepOnePlus Real-Time PCR System implementing TB Green Premix Ex Taq (Takara, Japan). Internal controls were GAPDH and small nuclear U6. Supplementary Table S1 demonstrates the primer sequences.

### Fluorescence in situ hybridization (FISH)

The probe of Cy3-labeled circVAMP3 (Supplementary Table S1) was synthesized by RiboBio (Guangzhou, China). FISH was exerted implementing a “Fluorescent in Situ Hybridization Kit” (RiboBio) following instructions. Nuclei were counterstained with DAPI, and the cells were assessed and visualized employing a confocal laser scanning microscope (Olympus, Japan).

### Vector construction and cell transfection

For stable knockdown of genes, 786-O cells were cultured and infected with lentivirus bearing short-hairpin RNA (shRNA) targeting circVAMP3, c-Abl, JAK2, or FGFR1 (GenePharma, Shanghai, China). The shRNA sequences are given in Supplementary Table S1. To stably overexpress circVAMP3 expression, we synthesized human circVAMP3 cDNA and cloned it into a pLVX-cir vector (GenePharma, Shanghai, China) for constructing overexpression plasmids; the control used an empty vector. The plasmid constructs were sequenced to confirm the success of the procedure. The plasmids were then transfected into HEK293T cells for packaging the lentivirus for ACHN cell infection.

All infected cells were cultured with 2 μg/mL puromycin for seven days, and the surviving cells were considered to be stably transfected and were used for further experiments.

### RNA pull-down assay

Specific biotin-labeled circVAMP3 (sense) and control (antisense) probes were prepared through RiboBio (Guangzhou, China) (Supplementary Table S1). RNA pull-down assessments were carried out as already explained [28]. In brief, cells (1 × 10^7^) were rinsed with chilled PBS, lysed in 500 μl Co-IP buffer, and incubated with 3 μg of the DNA oligo probes, for 2 h at ambient temperature. Streptavidin C1 magnetic beads (50 µl, Invitrogen) were added and incubated at ambient temperature for 1 h. Following rinsing with Co-IP buffer, the proteins were eluted, applied to SDS-PAGE, and the bands visualized using a “Coomassie Brilliant Blue staining kit” (KeyGEN, China) or immunoblotted. The circVAMP3 band was excised and subjected to mass spectrometry for analysis.

### Animal studies

The animal experiments were confirmed through the “Experimental Animal Care Commission” of Sun Yat-sen University. The processes executed in the current research were in compliance with the ethical benchmarks of the institutional ethics committee and with the “NC3Rs ARRIVE” protocols.

To establish RCC-bearing xenograft models, 4-week-old nude male BALB/c mice were obtained from Sun Yat-sen University Experimental Animal Center. All the animals were randomly grouped. A total of 5 × 10^6^ RCC cells transduced with either circVAMP3-sh1/2 or circVAMP3 were subcutaneously injected into the dorsal surface of the thighs. Tumors were measured each week and the volumes (mm^3^) were evaluated as “Volume = 0.5 × length × width^2^.” Five weeks later, mice were killed through CO_2_ inhalation, subsequently the tumors were excised, weighed, and analyzed with the aid of IHC.

### Statistics

The assessments were conducted at least three times, and the outcomes are represented as the mean ± SD. Statistical discrepancy between groups was scrutinized employing SPSS 20.0. Student’s *t*-test or ANOVA was applied for parametric variables. *P*-values lower than 0.05 were regarded statistically meaningful.

## Supplementary information

Supplementary Figure S1

Supplementary Figure Legends

Revised Supplementary Data S1

Supplementary Table S1

Reproducibility Checklist

Original image of WB (3-20)

## Data Availability

The datasets employed or/and scrutinized within the present investigation are accessible from the corresponding author on reasonable requests.
