# Supplementary figures and images for: Circular RNA circVAMP3 promotes aerobic glycolysis and proliferation by regulating LDHA in renal cell carcinoma
Source: Cell Death Dis. 2022 May 7;13(5):443. doi: 10.1038/s41419-022-04863-0 (PMC9079058; doi:10.1038/s41419-022-04863-0)

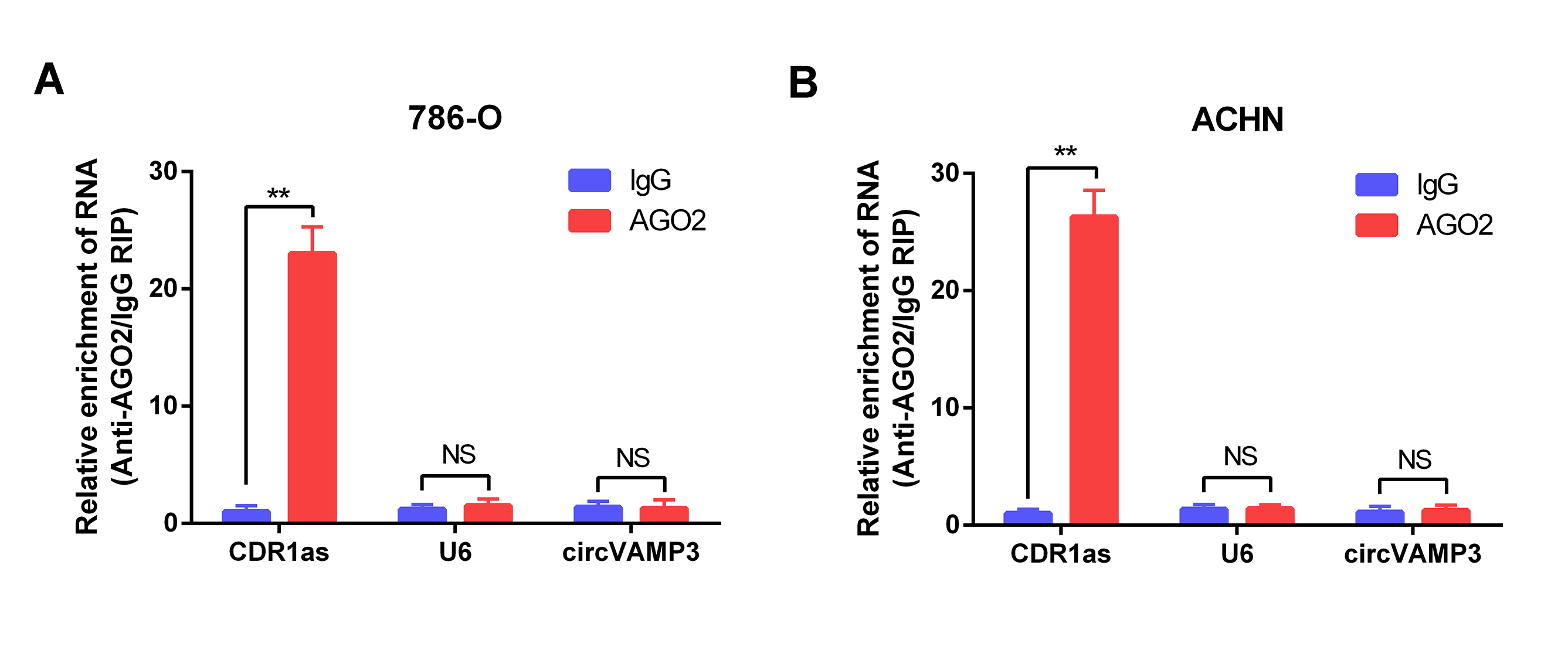

Supplement: Supplementary file 1 — Supplementary Figure S1 [file 41419_2022_4863_MOESM1_ESM.tif]
