# Supplementary material for: Circular RNA circVAMP3 promotes aerobic glycolysis and proliferation by regulating LDHA in renal cell carcinoma
Source: Cell Death Dis. 2022 May 7;13(5):443. doi: 10.1038/s41419-022-04863-0 (PMC9079058; doi:10.1038/s41419-022-04863-0)
Supplement: Supplementary file 2 — Supplementary Figure Legends [file 41419_2022_4863_MOESM2_ESM.docx]

**Supplementary Figure Legends**

**Supplementary Fig. S1 qRT-PCR analysis of RNA enrichment in the RIP assay using the anti-AGO2 antibody in 786-O (A) and ACHN (B) cells.** lgG: nonspecific control antibody; CDR1as: positive control; U1: negative control.
