# Supplementary material for: Circular RNA circVAMP3 promotes aerobic glycolysis and proliferation by regulating LDHA in renal cell carcinoma
Source: Cell Death Dis. 2022 May 7;13(5):443. doi: 10.1038/s41419-022-04863-0 (PMC9079058; doi:10.1038/s41419-022-04863-0)
Supplement: Supplementary file 3 — Revised Supplementary Data S1 [file 41419_2022_4863_MOESM3_ESM.docx]

**Supplementary Data S1**

**Supplementary materials and methods**

**Bioinformatics analysis**

circRNA microarray data of paired normal and ccRCC tissues were downloaded from the “NCBI GEO Datasets” (<http://www.ncbi.nlm.nih.gov/gds>, No. GSE108735). Normalized microarray data were investigated employing GEO2R following exerting applying log2 transformation. GEO2R permits comparing the original submitter-supplied progressed information (<https://www.ncbi.nlm.nih.gov/geo/geo2r/>).

**Actinomycin D assay**

The cells were incubated with medium containing 2 μg/ml Actinomycin D (Abcam, UK) to block transcription. Control cells did not receive Actinomycin D. Incubation with Actinomycin D was conducted for 8, 16, and 24 h, following which the cells were collected, and the RNA was assessed by qRT-PCR.

**Glucose uptake and lactate secretion**

Glucose contents were assayed with a “Glucose Colorimetric Assay Kit” (BioVision, USA) and lactate concentrations were evaluated by implementing a “Lactate Assay Kit” (BioVision), following the supplied protocols. Glucose and lactate levels were determined relative to those of the negative controls. In brief, cells at a density of 2,000 cells were seeded per well in a plate containing 96 wells. Cells were comprising starved in serum free cultivation milieu for 2 hours. Cells were subsequently rinsed by employing PBS and incubated with 100 μL Krebs-Ringer-Phosphate-HEPES (KRPH) buffer 2% BSA for 40 min for the evacuation of endogenous glucose, succeeded through 10 μL 2-deoxyglucose (10 mM) incubation for 20 min. Cells were accumulated with extraction buffer and processed for detecting glucose adsorption capability. The levels of glucose adsorption were appraised though OD at 412 nm in a microplate reader and normalized to cell number.

**Extracellular acidification rate (ECAR) and oxygen consumption rate (OCR)**

The glycolysis capacity and proton leak rate were assessed with a “Seahorse XF Glycolysis Stress Test Kit” and a “Seahorse XF Mitochondrial Stress Test Kit” (Agilent, USA), accordingly. In brief, 4×10^4^ cells were plated in the plates containing 96 wells and cultivated during the night hours. The cells were then washed by employing Seahorse buffer, and 25 mL each of 10 mmol/L glucose, 1 mmol/L oligomycin, and 100 mmol/L 2-deoxy-glucose were added for ECAR determination. For OCR analysis, 2 mmol/L glutamine, 1 mmol/L pyruvate, and 10 mmol/L glucose were used. The values of ECAR and OCR were assessed and normalized to cell number.

**CCK-8 assay**

Cellular viability was evaluated with a “Cell Counting Kit-8” (KeyGen, Nanjing, China). Approximately 3×10^3^ cells were plated in the plates containing 96 wells and assessed after 0, 24, 48, 72, and 96 h. Ten microliters of CCK-8 solution was placed in each well and incubated at 37℃ for 2 hours after which absorbances at 450 nm were read in a microplate reader (MD, USA).

**Colony-formation assay**

Cells were plated in the plates containing 6 wells at 1×10^3^ cells per well. Approximately 10 days later, the cells were fixed with 4% formaldehyde and visualized by crystal violet staining. Colonies with greater than 50 cells were measured microscopically. Three replicates were used for each.

**5-Ethynyl-2’-deoxyuridine (EdU) assay**

EdU proliferation assessments were accomplished employing an “EdU assay kit” (RiboBio, China) as previously described. The cells were incubated with 50 μM EdU for 2 h, followed by Apollo staining and DAPI counterstaining.

**RNA immunoprecipitation (RIP)**

RIP was executed applying the “EZ-Magna RIP Kit” (Millipore, USA). Cells (1 × 10^7^ cells) were collected and lysed in RIP lysis buffer and a single freeze-thaw cycle. Lysates were co-immunoprecipitated with an anti-LDHA antibody (Abcam, USA), and the RNA was measured by qRT-PCR. The negative control was mouse IgG, and U1 RNA was the non-specific control for qRT-PCR.

**Immunofluorescence**

Cells were grown on coverslips, fixed for 20 min in 4% paraformaldehyde, and treated with 0.1% Triton X-100 for 15 min. Following washing (PBS) and blocking (5% BSA in PBS, 30 min, 37 ℃), the cells were probed with anti-LDHA during the night hours at 4℃. Following rinsing, cells were probed with the appropriate secondary antibody for 1 h, sealed with DAPI-containing parafilm, and examined under confocal microscopy.

**Enzyme activity measurement**

LDH activity was discerned implementing an “LDH activity assay kit” (BioVision) in accordance with the manufacture’s protocols. Cells (1 × 10^6^ cells) were homogenized in cold assessment buffer, centrifuged, and the supernatant retained. One hundred microliter reaction mixtures containing 50 μl diluted cell lysate, 44 μl assessment buffer, 2 μl enzyme mix, 2 μl substrate mix, and 2 μl detection probe solution were included in 96-well plates. LDH activity was ascertained through evaluating the absorbances at 450 nm in a microplate reader.

**Western blotting**

Cells were lysed in RIPA buffer (KeyGEN, China) and the protein contents of the lysates assessed through a “BCA assay kit” (KeyGEN, China). The proteins were divided on 12% SDS-PAGE and electroblotted to PVDF. Following blocking 5% BSA for 1 h at ambient temperature, the membranes were probed with primary antibodies against LDHA (1:1000, Abcam), p-LDHA (Tyr10) (1:1000, Abcam), FGFR1 (1:500, Abcam), c-Abl (1:1000, Abcam), JAK2 (1:500, Abcam) or β-actin (1:1000, Abcam) during the night hours at 4°C. The membranes were subsequently incubated with corresponding secondary antibodies (1:5000, Abcam) for 1 hour. In the last step, the visualization of the bands was done by employing

ECL-plus western blotting detection reagents (BD Biosciences, USA). β-actin was employed as an internal standard.

**Co-immunoprecipitation (Co-IP)**

Co-IP was conducted as already reported [1]. Briefly, whole-cell lysates were incubated with antibodies against c-Abl (1:1000, Abcam), JAK2 (1:500, Abcam), FGFR1 (1:500, Abcam) or control lgG (1:1000, Abcam) with agitated rotation at 4℃ overnight. Western blotting was used for further analysis.

**GST pull-down assay**

The pull-down was conducted as already defined [2]. Glutathione beads were coated with GST or GST-fusion proteins (GST-LDHA and GST-lgG) in PBS and incubated with RCC cell lysates or recombinant FGFR1 protein in the reaction buffer. The material was incubated at ambient temperature for 1 h with rotation, centrifuged, and the pellet rinsed with reaction buffer three times. The proteins bound to the beads were eluted and resolved on SDS-PAGE followed by western blotting.

**Immunohistochemistry (IHC)**

Immunohistochemistry was performed as previously described [3]. Tissues were fixed in 4% formalin, paraffin-embedded, and sections (4 μm) were prepared. After blocking endogenous peroxide activity and non-specific protein binding, sections were incubated during the night hours at 4°C with primary antibodies. Following rinsing in PBS, the sections were incubated at 37°C for 1 h with HRP-polymer-conjugated secondary antibody, followed by DAB staining and hematoxylin counterstaining for 3 min. The slides were assessed by blinded investigators and the proportions of positive cells (0, 0%; 1, 1%‐25%; 2, 26%‐50%; 3, 51%‐75%; 4, 76%‐100%) and staining intensity (0: negative, 1: weak, 2: moderate, and 3: strong). The final IHC score was calculated through multiplying the intensity score and the percentage score.

**References**

1. Adhikary S, Sanyal S, Basu M, Sengupta I, Sen S, Srivastava DK, et al. Selective Recognition of H3.1K36 Dimethylation/H4K16 Acetylation Facilitates the Regulation of All-trans-retinoic Acid (ATRA)-responsive Genes by Putative Chromatin Reader ZMYND8. J Biol Chem. 2016; 291:2664-81.
2. Ji H, Ding Z, Hawke D, Xing D, Jiang BH, Mills GB, et al. AKT-dependent phosphorylation of Niban regulates nucleophosmin- and MDM2-mediated p53 stability and cell apoptosis. EMBO Rep. 2012; 13:554-60.
3. Xiao H, Wang J, Yan W, Cui Y, Chen Z, Gao X, et al. GLUT1 regulates cell glycolysis and proliferation in prostate cancer. Prostate. 2018; 78:86-94.
