# Supplementary material for: Circular RNA circVAMP3 promotes aerobic glycolysis and proliferation by regulating LDHA in renal cell carcinoma
Source: Cell Death Dis. 2022 May 7;13(5):443. doi: 10.1038/s41419-022-04863-0 (PMC9079058; doi:10.1038/s41419-022-04863-0)
Supplement: Supplementary file 4 — Supplementary Table S1 [file 41419_2022_4863_MOESM4_ESM.docx]

**Supplementary Table S1. The sequences used in this study are listed as follows.**

| **Gene** | **Sequence (5’-3’)** |
| --- | --- |
| **Primers for qRT-PCR** | |
| circVAMP3 Forward | TGGGCAATCGGGATTACTGT |
| circVAMP3 Reverse | TCAAATTGAGAAGCGCCTGC |
| mVAMP3 Forward | GTGGTGGACATAATGCGAGT |
| mVAMP3 Reverse | TGCAATTCTTCCACCAATAT |
| GAPDH Forward | CAATGACCCCTTCATTGACC |
| GAPDH Reverse | TTGATTTTGGAGGGATCTCG |
| U6 Forward | TTACCTGGCAGGGGAGATA |
| U6 Reverse | ACTACCACAAATTATGCAGT |
| U1 Forward | CGCTTCGGCAGCACATATAC |
| U1 Reverse | TTCACGAATTTGCGTGTCAT |
| CDR1as Forward | CGGGTCTTCCAGGAAATCCG |
| CDR1as Reverse | TCCGGAAGATGTGGATTGAC |
| **ShRNA oligo sequences** | |
| circVAMP3-sh1 | CCGGTATTTCCTCTTCAACTTGGCTCTCGAGAGCCAAGTTGAAGAGGAAATATTTTTG |
| circVAMP3-sh2 | CCGGTCATCGGTGGTGGACATAATGCTCGAGCATTATGTCCACCACCGATGATTTTTG |
| sh-c-Abl | CCGGCCCACATGGTAATGTCCTAGTCTCGAGACTAGGACATTACCATGTGGGTTTTTG |
| sh-JAK2 | CCGGCGTTTATCTAGGTCAAGAAGTCTCGAGACTTCTTGACCTAGATAAACGTTTTTG |
| sh-FGFR1 | CCGGCCATGTGTGACATTGAAGAGGCTCGAGCCTCTTCAATGTCACACATGGTTTTTG |
| scramble | CCGGCAACAAGATGAAGAGCACCAACTCGAGTTGGTGCTCTTCATCTTGTTGTTTTTG |
| **Oligonucleotides for FISH and RNA pull-down** | |
| circVAMP3 FISH | TCTTCATCATCATCATCATCGGTGGTGGACATAATGCGAGTTAAC-Cy3 |
| Sense probe for circVAMP3 pull-down | TCTTCATCATCATCATCATCGGTGGTGGACATAATGCGAGTTAAC-Biotin |
| Anti-sense probe for circVAMP3 pull-down | GTTAACTCGCATTATGTCCACCACCGATGATGATGATGATGAAGA-Biotin |
