# Supplementary material for: Circular RNA circVAMP3 promotes aerobic glycolysis and proliferation by regulating LDHA in renal cell carcinoma
Source: Cell Death Dis. 2022 May 7;13(5):443. doi: 10.1038/s41419-022-04863-0 (PMC9079058; doi:10.1038/s41419-022-04863-0)
Supplement: Supplementary file 6 — Original image of WB (3-20) [file 41419_2022_4863_MOESM6_ESM.docx]

**Original image for Figure 5C**


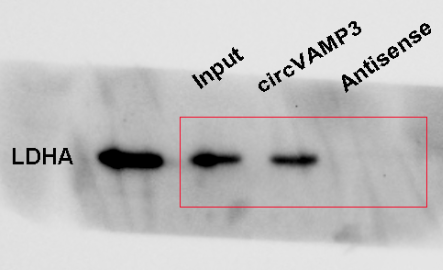


**37 kDa**

**Original image for Figure 5D**


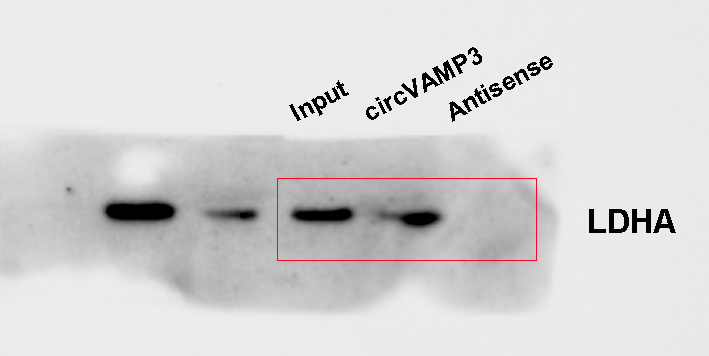


**37 kDa**

**Original image for Figure 5H**


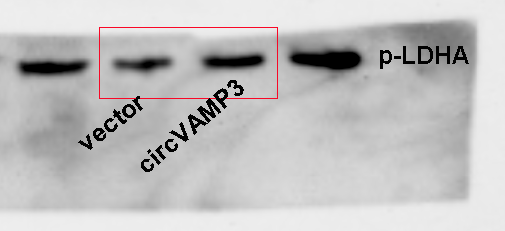

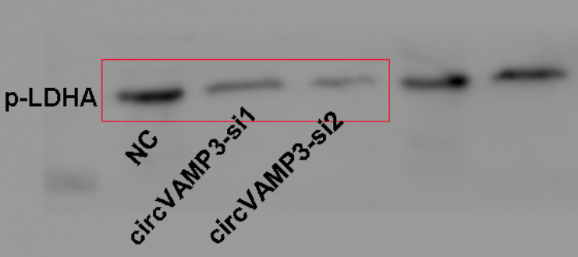


**37 kDa 37 kDa**


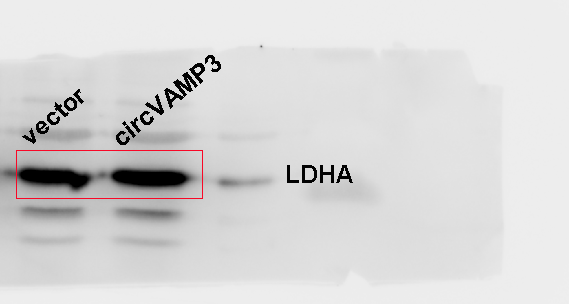

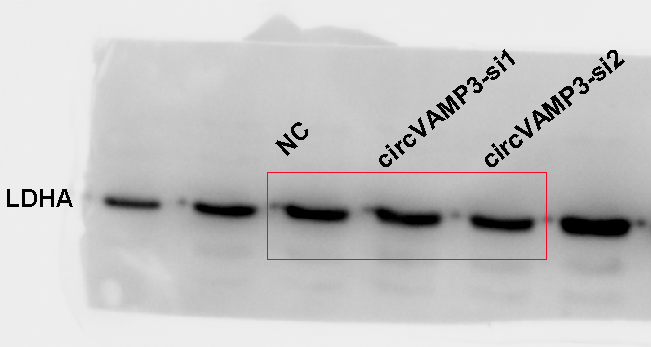


**37 kDa 37 kDa**


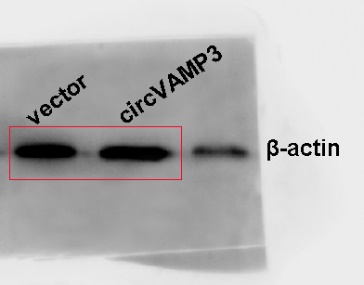

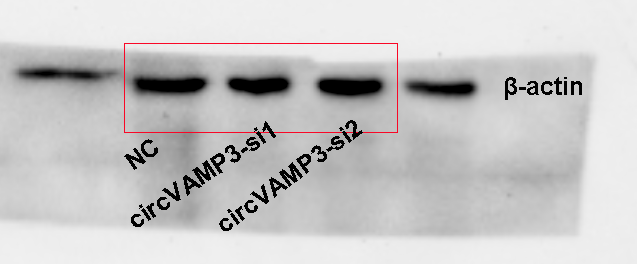


**42 kDa 42 kDa**

**Original image for Figure 6A**


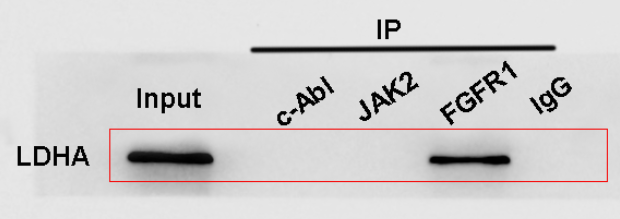


**37 kDa**


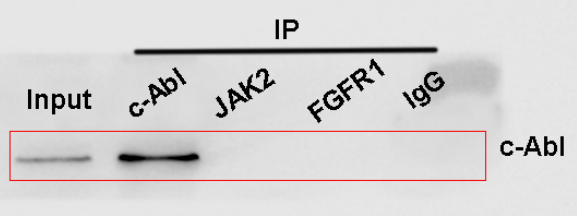


**120 kDa**


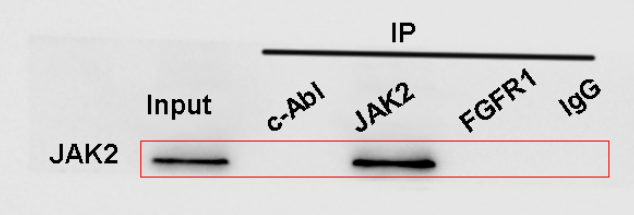


**131 kDa**


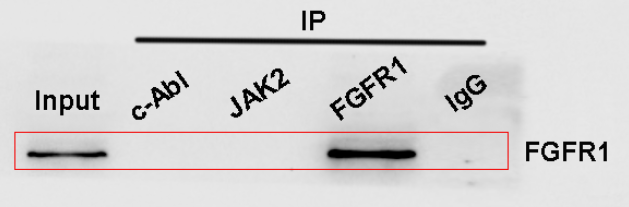


**120 kDa**


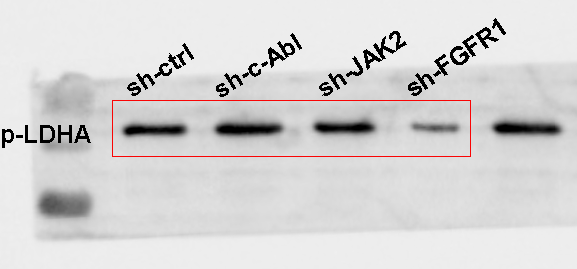
**Original image for Figure 6B**

**37 kDa**


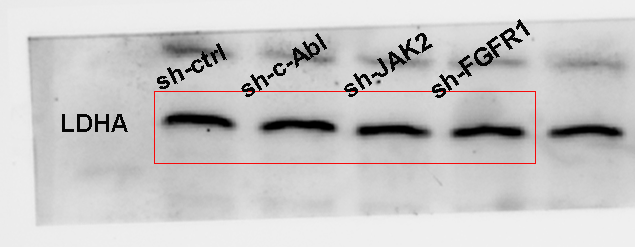


**37 kDa**


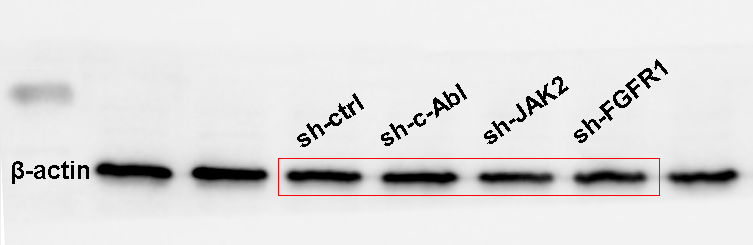


**42 kDa**


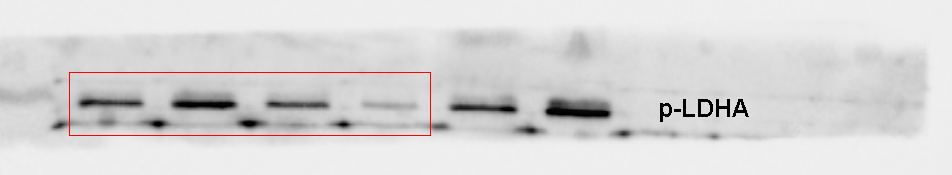
**Original image Figure 6C**

**37 kDa**


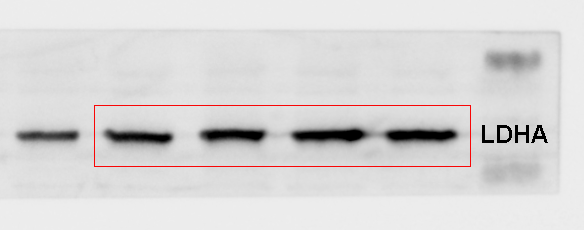


**37 kDa**


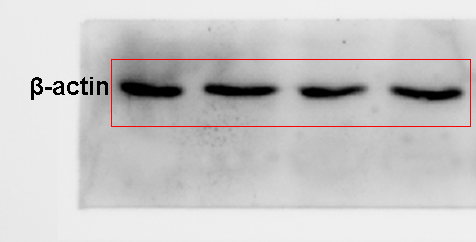


**42 kDa**

**Original image Figure 6D**


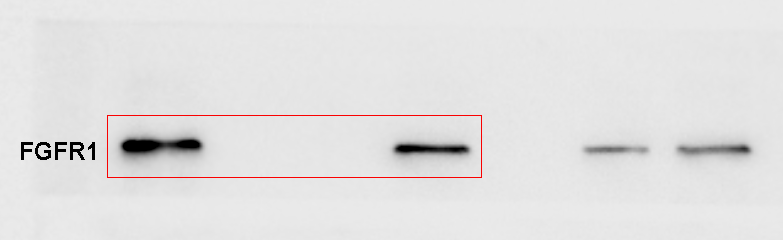


**120 kDa**

**Original image Figure 6E**


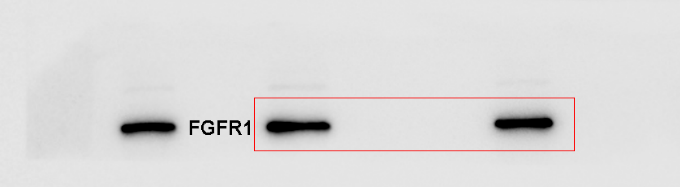


**120 kDa**


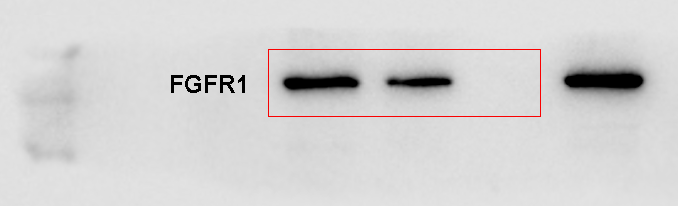
**Original image Figure 6F**

**120 kDa**

**Original image Figure 6G**


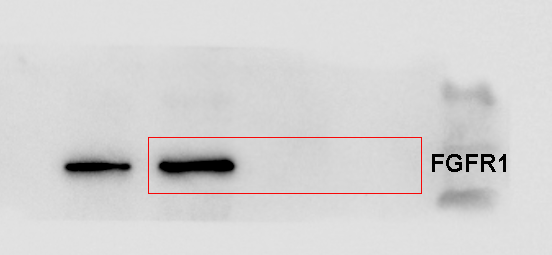


**120 kDa**

**Original image Figure 6H**


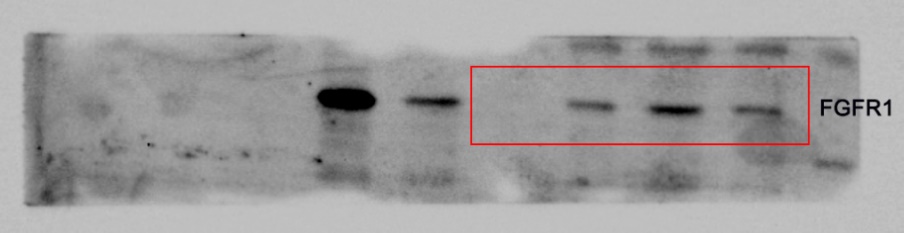


**120 kDa**


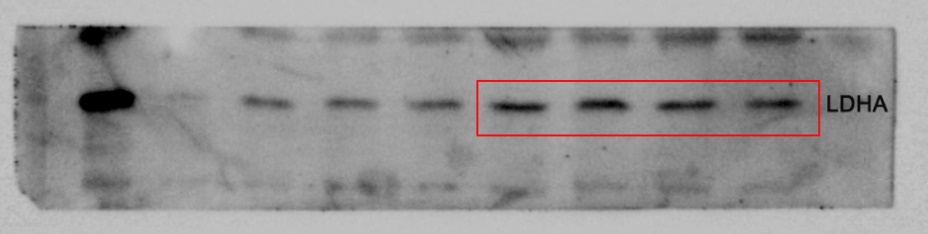


**37 kDa**
